# Children Cheat to Return a Favor

**DOI:** 10.1111/desc.70059

**Published:** 2025-08-24

**Authors:** Laura Tietz, Felix Warneken, Sebastian Grueneisen

**Affiliations:** ^1^ Faculty of Education Leipzig University Leipzig Germany; ^2^ Department of Psychology University of Michigan Ann Arbor USA

**Keywords:** cheating, dishonesty, interpersonal obligations, reciprocity, social cognition

## Abstract

Reciprocity is a cornerstone of human cooperation, motivating individuals to assist each other at a personal cost, resulting in mutual long‐term benefits. However, reciprocity can conflict with honesty norms, such as when returning favors to previous benefactors requires individuals to act dishonestly. The resulting moral dilemmas are difficult to navigate even for adults, yet developmental research has almost exclusively focused on the prosocial aspects of reciprocity. To explore the developmental origins of this conflict, we investigated 5‐ to 8‐year‐old children's evaluations of and engagement in prosocial cheating to return a favor. In Study 1, children evaluated protagonists in hypothetical scenarios who cheated to benefit others whom they did or did not owe a favor. Across ages, children condemned cheating and favored honesty over reciprocity from a third‐party perspective. In Study 2, children participated in two games in which they had the opportunity to cheat (by peeking behind a barrier or misreporting die‐rolling outcomes, respectively) to win prizes for an adult partner who had either previously shared a valued resource with them (reciprocity condition) or not (control condition). Across ages, children were more likely to benefit a partner by cheating when they owed them a favor than when they did not owe them a favor. These findings suggest that children as young as 5 are willing to disregard honesty in favor of reciprocating prosocial acts, highlighting the developmental origins of a tension between interpersonal obligations and moral norms.

**Summary:**

- In two studies, we investigated 5‐ to 8‐year‐old children's evaluations of and engagement in prosocial cheating and prosocial cheating to return a favor.
- From a third‐party perspective, children strongly endorsed rule compliance and condemned cheating, even when it helped someone else.
- When acting themselves, children cheated to benefit a partner, especially when the partner had previously done them a favor.
- The results reveal that reciprocity can override honesty norms in early childhood, shaping moral decision‐making.

## 1 Introduction

Humans engage in a wide range of cooperative activities: Many of our everyday interactions involve alternating acts of kindness (such as your neighbor feeding your cat during your yoga retreat and in return, you watering her plants while she is on vacation). This exchange of favors between two people over repeated interactions—direct reciprocity—is a key mechanism stabilizing cooperation between non‐kin, where taking turns paying the costs and receiving the benefits ensures long‐term mutual benefit for both interaction partners (Axelrod and Hamilton 1981; Nowak 2006; Trivers 1971).

Yet, reciprocity may not be inherently virtuous. While it promotes social cohesion, it can also motivate ethically questionable behaviors that undermine collective welfare, especially when reciprocal obligations conflict with moral norms. Imagine a civil servant helping a colleague meet a tight deadline by working overtime without compensation. In return, that colleague turns a blind eye when the civil servant improperly files private trips as business expenses. In situations like these, a conflict arises between the interpersonal obligation to reciprocate the favor and the moral obligation to uphold honesty norms. Such moral dilemmas are difficult to navigate and, in extreme cases, can foster corruption if individuals prioritize interpersonal obligations over ethical principles like honesty. Indeed, research has demonstrated that adults are oftentimes more dishonest in cooperative settings than when acting out of pure self‐interest, and this cooperative dishonesty has been attributed to feelings of commitment toward cooperation partners (Leib et al. 2021; Weisel and Shalvi 2015; Zickfeld et al. 2024).

However, while this phenomenon has received increasing attention in research with adults in the last decade, its developmental origins remain poorly understood. Specifically, the question arises: How do children navigate this kind of dilemma between honesty and reciprocity? A substantial body of research has investigated the emergence of reciprocal cooperation in early childhood, yet the primary focus has been on its positive, prosocial, and cooperative aspects. Existing studies have demonstrated that, starting from age 3, children reciprocate prosocial acts by sharing more resources with individuals who intentionally shared with them, compared to those who did not share (Warneken and Tomasello 2013) or provided benefits accidentally (Vaish et al. 2018). With increasing age, children's capacity for reciprocal behavior refines and becomes more strategic. By age 5, children selectively share more with someone who can later reciprocate (Grueneisen et al. 2023; Sebastián‐Enesco and Warneken 2015; Xiong et al. 2016), consistently engage in reciprocal resource exchanges with peers (House et al. 2013), and spontaneously develop turn‐taking strategies in collaborative tasks in which interaction partners’ interests are in conflict (Grueneisen and Tomasello 2017; Melis et al. 2016).

Importantly, children also come to understand reciprocity as a normative standard. In a study by Wörle and Paulus (2019), children were asked to evaluate individuals engaged in a sequential sharing task. While 3‐ to 4‐year‐olds generally favored prosociality and disliked selfishness, by age 5 to 6, children were selective based on whether the protagonists had a reciprocal obligation to return a favor: They only condemned individuals who failed to share with partners who had previously shared with them and not those who decided not to share with a previously selfish partner. In addition to this sensitivity to reciprocal obligations in third‐party interactions, children also uphold obligations arising from their own collaborative activities. Preschool children resist the temptation to abandon their collaboration partner when given the opportunity to defect (Kachel and Tomasello 2019; Koomen et al. 2020) and continue to work together to ensure that their partner receives a reward, even after they themselves have been rewarded (Hamann et al. 2012). Together, this work suggests that, from age 3 to 5, children become skilled reciprocal cooperators who are not only aware of others’ obligations but also recognize that commitments to cooperative partners apply to themselves.

At the same time, children also value honesty and disapprove of lying and cheating for selfish reasons (Marlow et al. 2023). Yet throughout early and middle childhood, children increasingly engage in those exact behaviors (Lee 2013). A classic paradigm used to investigate dishonesty in children is the temptation resistance paradigm, in which children are told not to peek at a toy in the experimenter's absence. While at age 3, the majority of children cheat but confess to having peeked (Talwar and Lee 2002), as capacities for inhibitory control and theory of mind mature with increasing age, children become more sophisticated at also lying to cover up their transgression (e.g., Talwar and Lee 2008).

Importantly, from preschool age, children's cheating is influenced by social cues. For example, telling children they have a reputation for being a “good kid” decreased cheating in 5‐year‐olds (Fu et al. 2016), and children aged 3 and 5 behaved more honestly when honesty was framed as a descriptive norm (i.e., typical behavior) or an injunctive norm (i.e., desired behavior) among their peers (Liu et al. 2022). Eliciting promises has been found to have a similar effect: Children from age 5 who promised not to cheat in the temptation resistance paradigm were indeed less likely to do so (Heyman et al. 2015).

Interestingly, recent studies indicate that children and adolescents engage in and approve of prosocial dishonesty. In a behavioral study, Sai et al. (2024) used a mind game in which 3‐ to 6‐year‐old children were asked to think of a number between 1 and 6 and then rolled a die. If the outcome matched their imagined number, children won a prize that they either got to keep (self‐interested condition) or give to an unknown child from a poor village (other‐interested condition). Children cheated both for their own benefit and to benefit another child. Zhao et al. (2019) used a card game version of the temptation resistance paradigm in which children could dishonestly report to have correctly guessed the number on a card. They found that cheating to benefit an unknown other child was more prevalent than selfish cheating, but only if the beneficial outcome was observable to others, suggesting that prosocial cheating was primarily motivated by a desire to signal prosociality. In a vignette study, 6‐ to 12‐year‐old children judged an individual who lied to increase a peer's reputation as more morally correct, trustworthy, and likable than an individual who told the truth, thereby decreasing the peer's reputation (Ahn et al. 2020). Relatedly, 15‐ to 18‐year‐old adolescents were more likely to predict that they would lie to protect a friend's reputation than to tell the truth that would expose their friend as a cheater (Shao et al. 2024). Hence, while younger children behave dishonestly to materially benefit others, adolescents also condone cheating to protect others’ reputation, suggesting that a diverse set of prosocial motives can elicit dishonesty.

However, while previous work has examined how children and adolescents navigate the tension between honesty and prosocial concerns, this work has not investigated whether the goal to reciprocate favors can compromise honesty. Answering this question has both theoretical and practical implications. First, if the motive to return favors encourages young children to cheat, this would call for an amendment to conceptions of reciprocity as a mechanism stabilizing cooperation already in childhood (House et al. 2013; Melis et al. 2016) and highlight its concurrent potential for eroding social norms and eliciting corrupt tendencies. Furthermore, addressing this issue has the potential to reveal an early‐emerging vulnerability, namely, that children's inclinations to return favors can be exploited, leading them to break rules they otherwise approve of in the service of benefiting others. This might spark new theorizing as well as reflections in educational settings—for instance, in the context of academic cheating (e.g., Daumiller et al. 2024)—about when, with whom, and in what contexts reciprocal cooperation should be encouraged and when it can result in adverse effects.

A phenomenon closely related to the current question is bribery, which also involves a conflict of honesty and reciprocal considerations. A series of studies on children's developing understanding of bribery demonstrated that from age 6 to 10, children increasingly perceived bribe acceptance as wrong in third‐party interactions and advised others against taking the bribe (Reyes‐Jaquez and Koenig 2021, 2022). When children were in a position to accept a bribe themselves as judges of a drawing contest, the majority refused to accept gifts from contestants offered on the condition of being selected (Reyes‐Jaquez and Koenig 2022, Study 2). This indicates that, by late childhood, children recognize that reciprocal motivations could undermine impartiality. However, the focus of this research was on children's evaluation of deliberate, illicit conspiracies where one person demands an illegitimate favor in return for benefiting the actor. By contrast, the current studies sought to investigate whether young children feel inclined to cheat to return a favor in the complete absence of explicit expectations, simply because they were the recipients of a prosocial act.

To this end, we first aimed to establish if children indeed perceive cheating to benefit others as wrong (Study 1). Given children's strong tendency to favor rule following (Rakoczy and Schmidt 2013), we hypothesized that, from a third‐party perspective, participants would condemn both prosocial cheating and prosocial cheating to reciprocate a favor.

The main study (Study 2) consisted of a behavioral experiment examining whether children do, in fact, cheat to return a favor. In two games—a guessing game modelled after the temptation resistance paradigm (Talwar and Lee 2002) and a child‐friendly version of the die‐rolling game by Fischbacher and Föllmi‐Heusi (2013)—participants had the opportunity to cheat to benefit a partner. We manipulated whether the partner had previously shared a resource with the child (reciprocity condition) or not (control condition). Based on previous developmental studies (Sai et al. 2024; Zhao et al. 2019), we expected children to cheat in both conditions. However, we hypothesized that children would cheat more in the reciprocity condition than in the control condition, as a result of the partner's previous prosocial act.

We tested these hypotheses with children aged 5 to 8 years because reciprocity, dishonesty, as well as children's balancing of honesty and other social motivations undergo substantial development over this period (Chernyak et al. 2019; Grueneisen and Warneken 2022; Lee 2013). While we did not have clear predictions regarding the effects of age, we aimed to test for potential developmental shifts in how children reconcile honesty and reciprocity norms.

## 2 Study 1

The goal of Study 1 was to investigate how children evaluate individuals who cheat to benefit others or to return a favor. We hypothesized that children would evaluate cheating to benefit others more negatively than rule compliance, regardless of whether the cheater had previously received a favor or not. We also hypothesized that children would be more inclined to advise others not to cheat than to break the rules. Study 1 was preregistered on AsPredicted (https://aspredicted.org/xgcc‐fjbw.pdf).

### 2.1 Method

#### 2.1.1 Participants

Forty‐eight children (24 female) aged 5 to 8 years were included (24 female; age range: 61 to 106 months; *M* = 83.12 months, SD = 13.47 months). One additional child participated but was excluded due to experimenter error. In a power analysis using G*Power (Faul et al. 2007), a sample size of *n* = 44 was determined to detect a within‐subject effect of *dz* = 0.54 with a 90% power and an α‐level of *p* = 0.05. Effect size estimates were derived from pilot data. To achieve an equal distribution of age groups across conditions, four participants were added.

All children participated at their respective childcare centers in an urban environment in Leipzig, a medium‐sized city in Germany. Parental written consent and children's verbal assent were required for participation. No data on participants' community of descent or socioeconomic status were collected, but the sample was drawn from a population in which 69% of 6‐ to 10‐year‐old children are ethnic German and 31% come from migrant families, encompassing a wide range of socioeconomic backgrounds (Stadt Leipzig 2024).

#### 2.1.2 Design and Procedure

In a 2 × 2 mixed design, participants were randomly assigned to either the reciprocity or the control condition (between‐subjects factor), with age and gender balanced between conditions. Each child completed two vignettes with a different set of protagonists—one involving cheating and one involving rule compliance (within‐subjects factor). Vignette order and the set of protagonists were counterbalanced between participants.

**FIGURE 1:**
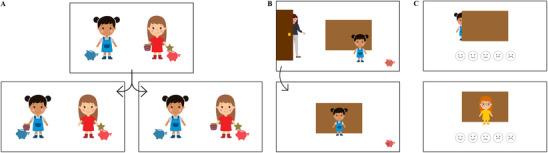
Key vignette slides. (A) Reciprocity manipulation: One child wins two stickers and subsequently shares one with the main protagonist (reciprocity condition, left panel), or does not share (control condition, right panel). (B) The protagonist is left alone before making a guess, and children stated what she should do (measure 1: advice). (C) The protagonist peeks (cheating vignette) or does not peek (rule compliance vignette), and children rated this behavior (measure 2: evaluation). Protagonists are edited versions of images from Vecteezy.com.

Children participated individually in a quiet room. A female experimenter showed children vignettes on a laptop. Vignettes were illustrated with cartoon characters and props in PowerPoint (see Figure 1). All vignettes featured a pair of child protagonists named according to the color of their clothing (e.g., Blue and Red) and an adult game master (all gender‐matched to the participant). First, a first player won two stickers in a game that they either spontaneously shared with the main protagonist (reciprocity condition) or not (control condition). The first player then left the room, and the protagonist played two rounds of a game in which they had to guess which of five animal figures was put on a cup behind a barrier. In the first round, they guessed correctly, thus winning a sticker for themselves. If the protagonist guessed correctly again, the first player would also win a sticker. The protagonist was then left alone and had the opportunity to cheat by peeking behind the barrier without being caught. Children were asked whether they thought the protagonist should peek and why.

Irrespective of children's response, the protagonist then either cheated (cheating vignette) or did not cheat (rule compliance vignette), and children were asked to rate their behavior by pointing on a five‐point Likert scale ranging from very good to very bad, illustrated with smiley faces. Children were also asked why they thought the protagonist had (or had not) cheated. The protagonist then guessed correctly, and the first player won a sticker.

Four control questions were included in each vignette to ensure children paid attention and understood all relevant aspects—that is, who had just won stickers, whether the first player had shared (asked once in the beginning and once in the end), and what would happen if the protagonist guessed incorrectly. Only one participant answered one question incorrectly and was corrected.

#### 2.1.3 Coding and Analyses

Sessions were videorecorded, and children's answers were coded from video by the first author. An additional coder re‐coded 12 randomly selected videos (i.e., 25% of the sample). Interrater reliability was perfect (*κ* = 1 for all variables).

All analyses were conducted in R Studio Version 4.4.1 (R Core Team 2024). Preliminary analyses confirmed that gender or the order in which vignettes were presented did not significantly influence children's answers. Hence, these variables were not included in the main analyses.

To analyze children's evaluations, we fitted linear mixed models (LMMs). Condition (reciprocity vs. control), age (in months), and vignette content (cheating vs. rule compliance) were entered as fixed effects, while subject number was entered as a random effect. Counter to our preregistration, we did not include random slopes, as including them led to model convergence issues.

As there was hardly any variation in children's advice to the protagonist, we only report descriptive results (rather than the preregistered mixed effects models).

To analyze children's explanations for why the protagonist might have peeked, we fitted generalized linear models (GLMs) with binomial error structure. The dependent variable was whether children indicated that the protagonist cheated to benefit the other child (e.g., “So that Green can have a sticker as well.”). We included age (in months), condition, and their interaction as predictors.

We always first compared the full model containing all relevant predictors and their interactions to a null model containing no predictor (in case of GLMs) or only the random effect (in case of LMMs) using a likelihood ratio test. When this comparison was significant, we continued with hypothesis‐driven tests of individual predictors.

In addition to frequentist model comparisons, we used Bayes factors (BF; not preregistered) to evaluate the strength of evidence for one model over the other using the *brms* package (Bürkner 2017) in combination with the *bridgesampling* package (Gronau et al. 2020). Our interpretation follows conventional BF benchmarks (BF_10_ = 1–3: anecdotal; BF_10_ = 3–10: moderate; BF_10_> 10: strong evidence).

### 2.2 Results

#### 2.2.1 Evaluations of Protagonist Behavior

**FIGURE 2:**
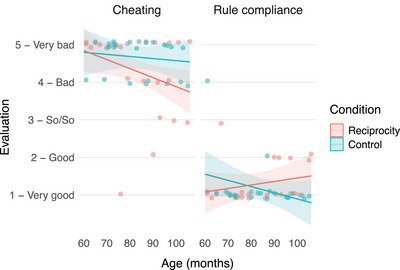
Children‘s evaluations of cheating and rule compliance in the reciprocity and the control condition (model plot including raw data points). Lines represent model estimates, shaded areas represent 95% confidence intervals.

The full‐null model comparison revealed a significant combined effect of the predictors on children's evaluations, *χ* ^2^(7) = 181.24, *p* < 0.001 (LMM). Further model comparisons revealed a significant three‐way interaction between vignette content, condition, and age, *χ* ^2^(1) = 4.18, *p* = 0.041 (see Figure 2).

Subsequently, we analyzed the influence of age and condition separately for the different vignette contents (for an alternative follow‐up analysis of the three‐way interaction examining the effect of age and vignette content in each condition, see the Supporting Information). Children's evaluations of cheating were not significantly influenced by age, condition, or their interaction, indicating that, across conditions and age groups, children evaluated cheating similarly negatively (*M* = 4.48, SD = 0.87; *bad—very bad*).

There was a significant interaction effect of age and condition on children's evaluation of rule compliance, *χ* ^2^(1) = 1.41, *p* = 0.037. Children in the control condition evaluated rule compliance slightly more positively with increasing age, although this effect did not reach significance (*b* = −0.02, SE = 0.01, *t*(44) = −1.76, *p* = 0.085). In the reciprocity condition, age did not significantly affect evaluations (*b* = 0.01, SE = 0.01, *t*(44) = 1.1, *p* = 0.279). Overall, all but two participants considered rule compliance *good* or *very good* (*M* = 1.23, SD = 0.59).

#### 2.2.2 Advice to the Protagonist

Only five children advised the protagonist to peek in one of the two vignettes (three in the reciprocity condition), and no child recommended peeking in both vignettes.

#### 2.2.3 Exploratory Analyses: Prosocial Motivations for Cheating

Overall, 17 children (35.4%) spontaneously referenced a prosocial motivation for the protagonist's cheating (see the Supporting Information for a more detailed categorization of explanations). The full‐null model comparison showed a significant combined influence of age and condition on children's prosocial explanations, *χ* ^2^(3) = 26.01, *p* < 0.001 (GLM). Further model comparisons revealed a significant interaction between condition and age, *χ* ^2^(1) = 4.16, *p* = 0.041. With age, children were more likely to recognize a prosocial motivation for cheating in both conditions (reciprocity condition: *b* = 0.09, SE = 0.04, *z* = 2.26, *p* = 0.024; control condition: *b* = 0.32, SE = 0.15, *z* = 2.14, *p* = 0.032). However, in the reciprocity condition, children started to recognize a prosocial motivation at a younger age (range: 74–106 months) than children in the control condition (range: 90–105 months).

#### 2.2.4 Bayesian Approach

Contrary to frequentist model comparisons, Bayesian model comparisons regarding vignette evaluations indicated strong evidence for the main effects model over the two‐way interactions model (BF = 176.09). With regards to prosocial explanations, Bayesian model comparisons yielded analogous conclusions to the frequentist model comparisons, indicating anecdotal evidence for the two‐way interactions model over the main effects model (BF = 2.74). For more details, see the Supporting Information.

Hence, in line with our hypothesis, children across all ages were opposed to both forms of cheating while endorsing rule compliance. However, older children demonstrated a more nuanced understanding of different competing motivations in the scenarios. With increasing age, children were more likely to spontaneously acknowledge potential prosocial motivations for the transgressions. These prosocial explanations were more frequent and emerged at an earlier age in the reciprocity condition, suggesting that children may be especially sensitive to prosocial obligations arising from interpersonal favors. Nevertheless, children clearly favored honesty and rule compliance over cheating to benefit others, at least when evaluating hypothetical scenarios as a neutral third party.

## 3 Study 2

Study 1 confirms that children condemn cheating, even if it is aimed at benefiting others or returning a favor. However, children in this study were not personally involved; they did not owe someone a favor and had the opportunity to cheat to settle their debts. Previous research has shown that from preschool age, children readily engage in reciprocal interactions (House et al. 2013; Vaish et al. 2018), recognize the normative aspects of reciprocity in third‐party interactions (Wörle and Paulus 2019), and actively adhere to obligations arising from collaborative activities with others (Hamann et al. 2012; Kachel and Tomasello 2019). Moreover, children's judgments of what one should do do not always match their own behavior (Smith et al. 2013). The main goal of Study 2 was, therefore, to investigate children's inclination to break the rules for the benefit of someone else, when that person either had or had not previously done them a favor. We hypothesized that children would be more likely to cheat to benefit someone else when they owed the person a favor than when they did not. Study 2 was preregistered on AsPredicted (https://aspredicted.org/mwfj‐pvhr.pdf, https://aspredicted.org/ct89‐ss2y.pdf).

### 3.1 Method

#### 3.1.1 Participants

A total of 184 children (92 female) aged 5 to 8 (age range: 60–107 months; *M* = 83.82 months, SD = 13.82 months), drawn from the same population as children in Study 1, were included. Fourteen additional children were tested but excluded due to experimenter error (2), because they failed comprehension checks (8), were too agitated or upset to complete the session (3), or cheated in the presence of the experimenter (1).

In a first step, we conducted Study 2 with 92 children aged 7 and 8. A simulation‐based power analysis using the R package *simr* (Green and MacLeod 2016) based on pilot data from 20 children indicated that a sample size of *n* = 138 would be necessary to detect a main effect of condition on cheating in the guessing game with a power of 80%. Due to constraints in participant recruitment, we adopted a sequential data collection and testing approach with adjusted α‐levels of *p* = 0.039 (Sagarin et al. 2014). After testing two‐thirds of the sample (*n* = 92), we conducted our main analysis and found *p*‐levels of *p *< 0.039 for the full‐null model comparison, hence stopping data collection, as preregistered. As a second step, we resumed data collection with 92 children aged 5 and 6.

Overall, 33.7% of children were tested in the laboratory, and the other 66.3% were tested at their respective childcare centers in Leipzig, Germany. Parental written consent and children's verbal assent were required for participation.

#### 3.1.2 Design and Procedure

In a between‐subjects design, participants were randomly assigned to either the reciprocity or the control condition, with age and gender balanced between conditions. We tested an equal percentage of children per condition in the lab and in childcare centers.

Children participated individually in a quiet room. A female experimenter (E) narrated the vignettes and acted as the game master, and a female research assistant acted as children's game partner (P).

##### 3.1.2.1 Reciprocity Understanding

In a pretest assessing the importance children ascribe to reciprocity in third‐party interactions, participants were presented with two short vignettes on paper sheets in a fixed order. Vignettes consisted of cartoon‐like props and characters named according to the color of their clothing (e.g., Purple and Red). In Vignette 1, a girl (Purple) shared her sandwiches with another girl (Pink). The following week, Purple forgot her lunch at home, and children were asked who should share their sandwiches with her—Pink or one of two neutral bystanders (Orange, Turquoise)—and why. In Vignette 2, a boy (Red) helped another boy (Blue) tidy up his room so Blue could join their peers at the playground. The following week, Red was not allowed to go to the playground until he cleaned up his room, and children were asked to indicate who should help him—Blue or one of the other kids (Green, Yellow)—and why. We recorded whether children picked the child who had previously shared with or helped the protagonist, respectively.

##### 3.1.2.2 Cheating Test 1: Guessing Game

**FIGURE 3:**
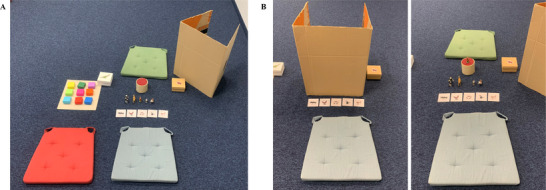
Materials and set‐up of cheating test 1 (guessing game). (A) General set‐up. Left: box game that the partner played. Right: guessing game that children played. (B) Guessing game trial from the child's perspective.

After inviting P to sit next to the child, E showed them some dinosaur and animal stickers and asked which they liked more. Once children had made a choice, P expressed that she liked both kinds equally. Next, E introduced two games: the box game and the guessing game (see Figure 3). The box game consisted of a set of differently colored opaque boxes arranged on a cardboard pad, and players could win the stickers that the child indicated liking more (henceforth: the *[preferred]* stickers). The guessing game consisted of five hard plastic animal figurines, a cardboard cup, and a cardboard barrier, and players could win the stickers the child indicated liking less (henceforth: the *[unpreferred]* stickers). E drew a lot, which decided, apparently at random, that the child would play the guessing game and P would play the box game.

E then explained to P that each box contained either two or zero stickers and that, in each round, P could pick two boxes and keep any stickers she found inside (unbeknownst to the child, boxes were prearranged so that P won two stickers in each round). Next, E explained to children that in the guessing game, she would place one of the five animals on the cup behind the barrier, and children had to guess which animal it was. Each round consisted of three trials. If children guessed correctly once, they would win a sticker. If they guessed correctly twice, P would also win a sticker. P then asked, “But can't [name of child] just peek behind the barrier and see which animal it is?” to which E responded that this would constitute cheating. This was done to ensure that children knew that peeking was indeed a rule violation.

P and the child played their respective game alternatingly, for a total of three rounds. First, P won two stickers in the box game. In the reciprocity condition, P then stated: “Hm, you really like the *[preferred]* stickers. I really like them as well! But you can't win any of the *[preferred]* stickers in your guessing game…You know what, I'll share my stickers with you. Now you also have a *[preferred]* sticker.” In the control condition, P kept the stickers to herself. Note that the rules of the box game were that P could keep any stickers she won, hence her decision to share in the reciprocity condition was entirely voluntary and spontaneous, while her decision to keep the stickers in the control condition was in line with the rules of the game.

In both conditions, P then left the room under a pretext, and E asked three comprehension check questions (i.e., how many guesses the child had, and how many times they had to guess right for them and for P to win a sticker). Incorrect answers were corrected. If children answered more than one question incorrectly, E asked an additional question right before the first test trial (i.e., who would win a sticker if they guessed correctly now). Children who answered this additional question incorrectly were excluded from all analyses.

During the first two trials of each round, the game was predetermined so that children guessed correctly and incorrectly once each, thus winning a sticker for themselves. Before children could guess a third time, E said: “Oh, I have to go get P, it's almost her turn again…I'll be back with P in one minute. When we get back, you can guess which animal it is. And if you guess correctly, P will also get a sticker. I'll be back in one minute!”

The dependent variable was whether children peeked behind the barrier in E's absence and subsequently guessed correctly. E could observe whether children peeked from outside the room via a smartphone app connected to a hidden camera. After 60 seconds, E and P returned, and children made their guess. In case children guessed correctly by chance (i.e., without peeking), E surreptitiously exchanged the animal, so that P only received a sticker if children cheated. This was done to ensure the same outcome for all children who did not cheat.

The procedure for all three rounds was identical except that we varied whether it was the first or the second trial during which P picked a box containing stickers and children guessed correctly, respectively. Before the third round, E announced that this was the final round.

##### 3.1.2.3 Cheating Test 2: Die‐Rolling Game

A coin toss determined—ostensibly at random—that the child rather than P would play the die‐rolling game. This was done to provide children with a valid reason why they, and not P, would play. In the game, children rolled a die behind a barrier. The die had an X on four sides and a dot on two sides. Children completed six die‐rolls in total, circled each outcome on a separate sheet of paper, and passed them to E. In die‐rolls 1 to 3–the main trials of interest—P was awarded a sticker for every dot children reported to have rolled. In die‐rolls 4 to 6, children were awarded a sticker for every dot they reported to have rolled. Only children could observe the true outcomes of the die rolls, so they could cheat by misreporting the outcomes.

Before children played, E reiterated that P had previously shared her stickers (reciprocity condition) or repeated who had won which stickers (control condition). Children were asked seven comprehension check questions before the die‐rolling game (i.e., how many times they would roll the die, who would win stickers in the first round and how many, depending on the outcome reports, and whether E could observe their die‐rolls). E corrected any incorrect answers, and children were asked those questions again. Children who answered any question incorrectly twice were excluded from analyses of the die‐rolling game.

#### 3.1.3 Coding and Analyses

The first author coded children's behavior from video. Two additional coders independently coded 25% of the sample (23 videos per coder). Inter‐rater reliability was almost perfect for children's vignette responses (κ = 0.99) and die‐rolling reports for the child (κ = 0.94), and perfect for children's cheating in the guessing game (κ = 1) and die‐rolling reports for the partner (κ = 1). In cases of coder disagreement, the first author's codings were used.

All analyses were conducted in RStudio Version 4.4.1 (R Core Team 2024). Gender, testing location (childcare centers vs. lab), or game partner (one of two female research assistants) were only included in the main analyses if preliminary analyses indicated a significant influence on the respective dependent variable.

For our main analysis, we fitted generalized linear mixed models (GLMMs) with binomial error structure and children's cheating in the guessing game as the dependent variable. Condition (reciprocity vs. control), age (in years), and trial (1 to 3) were entered as fixed effects, and participant number was entered as a random effect. As including age in months and random slopes led to convergence issues, we used age in years and dropped random slopes from these models.

In the die‐rolling game, three children were excluded because they failed the comprehension checks (2) or were unwilling to participate (1). We used Wilcoxon signed‐rank tests to compare the mean number of reported dots to chance (separately for each condition) and a Wilcoxon rank‐sum test to compare the mean number of reported dots between conditions. We fitted a GLMM including the same predictors as for the guessing game, but with reporting a successful outcome (i.e., a dot) for the partner (yes/no) as the dependent variable. We included participant number as a random effect, as well as random slopes of trial nested within participant number.^1^

In a third GLMM analysis, we pooled data from the two cheating tests and analyzed if condition, age (in years), or game (guessing game vs. die‐rolling game) influenced whether children produced a reward for the partner. We included participant number as a random effect, as well as random slopes of game nested within participant number.

In an exploratory analysis, we analyzed children's dot reports for themselves using the same non‐parametric testing approach as in the analysis of children's dot reports for the partner (see the Supporting Information for more details).

For all GLMMs, we first compared the full model containing all relevant predictors and their interactions to a null model containing only the random effects using a likelihood ratio test. When this comparison was significant, we continued with hypothesis‐driven tests of individual predictors.

To analyze children's vignette responses, we tested whether their choice of who should share/help differed from chance using Wilcoxon signed‐rank tests (separately for each vignette). We also preregistered an exploratory analysis investigating whether children's vignette responses predicted children's tendency to cheat. However, due to low variation in vignette responses, especially among older children, we report this analysis in the Supporting Information.

Again, we additionally compared models using a Bayesian approach to evaluate the strength of evidence for the competing models using the same approach as in Study 1 (not preregistered).

### 3.2 Results

#### 3.2.1 Cheating Test 1: Guessing Game

**FIGURE 4:**
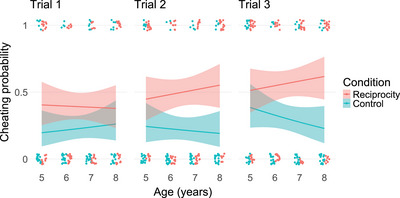
Effects of condition and age on children's cheating in the guessing game, separately for each trial (raw data plots including raw data points). Shaded areas represent 95% confidence intervals.

The full‐null model comparison indicated a significant combined effect of condition, age, and trial on children's cheating in the guessing game, *χ* ^2^(7) = 42.42, *p* < 0.001 (GLMM). Further model comparisons revealed a significant three‐way interaction, *χ* ^2^(1) = 3.97, *p* = 0.046. Subsequently, we analyzed the effect of condition and age for each trial separately (see Figure 4).

These analyses revealed main effects of condition in all three trials (trial 1: *χ* ^2^(1) = 5.77, *p* = 0.016; trial 2: *χ* ^2^(1) = 16.31, *p* < 0.001; trial 3: *χ* ^2^(1) = 12.91, *p* < 0.001). Hence, across all ages and trials, children in the reciprocity condition were significantly more likely to cheat than children in the control condition.

Although not statistically significant, there were descriptive differences in children's cheating depending on their age in interaction with trial. While the condition difference existed in all age groups, it was especially pronounced in 7‐ and 8‐year‐olds and increased as the game progressed. In contrast, 5‐year‐olds were comparatively more likely to cheat in the control condition, while 6‐year‐olds displayed the lowest cheating rates in both conditions. For a more detailed additional analysis of effects of condition and trial in the four age groups, see the Supporting Information.

#### 3.2.2 Cheating Test 2: Die‐Rolling Game

Children's reports of dots for the partner significantly differed from chance in the reciprocity condition (*M* = 1.27, SD = 0.95; *V* = 1001, *p* = 0.007), but not in the control condition (*M* = 1.04, SD = 0.74; *V* = 611, *p* = 0.575). Hence, children in the reciprocity condition generally over‐reported dots for the partner (i.e., cheated), whereas in the control condition, there was no evidence for cheating. However, when comparing conditions directly, there was no significant difference (*W* = 3630.5, *p* = 0.161).

Likewise, comparing the full model to the null model did not indicate a significant combined effect of condition, age, and trial on children's reports of dots for the partner, *χ* ^2^(7) = 6.88, *p* = 0.442 (GLMM).

#### 3.2.3 Analysis of Both Cheating Tests Combined

The identity of the game partner had a significant influence on children's generation of a reward for the partner (*χ* ^2^(1) = 4.59, *p* = 0.032), and was thus included in the main analysis as a control variable.

The full‐null model comparison revealed a significant combined effect of condition, age, and game on children's behavior (*χ* ^2^(7) = 32.83, *p* < 0.001). Further analyses revealed a significant interaction between condition and game (*χ* ^2^(1) = 14.74, *p* < 0.001; GLMM), corroborating the results of the individual game analyses. In the guessing game, cheating was more prevalent in the reciprocity condition than in the control condition (*χ* ^2^(1) = 19.48, *p* < 0.001), but this condition difference did not reach significance in the die‐rolling game (*χ* ^2^(1) = 3.43, *p* = 0.064).

#### 3.2.4 Exploratory Analysis: Children's Die‐Rolls for Themselves

Children's reports of dots for themselves significantly differed from chance both in the reciprocity condition (*M* = 1.62, SD = 0.97; *V* = 1633, *p* < 0.001) and the control condition (*M* = 1.49, SD = 1.02; *V* = 1517, *p* < 0.001), but did not differ significantly between conditions (*W* = 3802, *p* = 0.388). This indicates that the extent to which children cheated for themselves did not differ between conditions in the die‐rolling game. Furthermore, children in both conditions reported significantly more dots for themselves than for the partner (reciprocity condition: *W* = 3218, *p* = 0.013; control condition: *W* = 5164, *p* = 0.002).

#### 3.2.5 Reciprocity Understanding

In their vignette responses, children were more likely than chance to pick the protagonist who had been the recipient of a prosocial act as the person who should provide assistance (sharing: 86.4%, *V* = 16695, *p* < 0.001; helping: 84.2%, *V* = 16585, *p* < 0.001), reflecting a clear understanding of the reciprocity norm.

#### 3.2.6 Bayesian Approach

Bayesian model comparisons yielded analogous conclusions to frequentist model comparisons with regards to the guessing game (i.e., moderate evidence for the full model over the reduced model, BF = 7.77, and strong evidence for the full model over the main effects model, BF = 33.49) and the die‐rolling game (i.e., moderate evidence for the null model over the full model, BF = 5.68). Model comparisons for the analysis of both cheating tests combined resulted in several warning messages and are thus only reported in the Supporting Information.

Hence, in support of our main hypothesis, 5‐ to 8‐year‐old children were more inclined to cheat for the benefit of someone who previously shared a valued resource with them than someone who had not. This pattern tended to be more pronounced in older children of the sample. These findings indicate that children are willing to violate honesty norms in favor of reciprocity concerns.

## 4 General Discussion

Across two studies, we investigated how 5‐ to 8‐year‐olds evaluated individuals who cheated to benefit others and whether children themselves would be more likely to cheat on behalf of individuals to whom they owed a favor. Study 1 showed that, across the studied age range, children endorsed honesty and condemned cheating in third‐party interactions, even when the cheater did not act for selfish reasons but to benefit someone else. Yet, Study 2 revealed that when children were personally faced with the conflict between honesty and reciprocity, many prioritized returning the favor over playing by the rules.

This divergence between children's judgments in hypothetical scenarios and their real‐life decisions reveals a knowledge‐behavior gap: Children endorse moral principles in their evaluations but do not necessarily apply them in their actions. Knowledge‐behavior gaps have been documented in other contexts such as sharing and fairness (Blake 2018; Blake et al. 2014; Smith et al. 2013). However, the discrepancy between children's hypothetical norm endorsement and their actual behavior typically decreases by age 7 to 8 in fairness contexts, as children learn to apply their judgments of others’ actions to themselves. By contrast, children's cheating to return a favor tended to increase with age while their third‐party evaluations remained largely consistent, suggesting different mechanisms (for a similar pattern of results in adolescents regarding their predictions about reporting others’ stealing, see Soter et al. 2025). The current results indicate an early‐emerging and persisting flexibility in children's balancing of conflicting norms depending on the degree of personal involvement and highlight that reciprocity holds the potential to undermine children's honesty in personally relevant situations.

Why do children choose to cheat when norms of honesty and reciprocity conflict? One possibility is that children do not perceive other‐oriented cheating as a transgression. However, this account seems unlikely as children's answers in Study 1 confirmed that they understand honesty as a norm that applies across contexts and despite conflicting prosociality or reciprocity concerns. Instead, context‐specific motivations emerging in personally relevant situations seemed to prompt children to disregard honesty norms and cheat against their better judgment. In the following, we consider several possible mechanisms that may have driven this effect.

One possibility is that being the recipient of a prosocial act may have instilled a sense of indebtedness or obligation in children, prompting them to reciprocate the favor even at the expense of breaching the game rules. This interpretation aligns with findings in adults who similarly violate honesty norms in favor of living up to interpersonal commitments (Weisel and Shalvi 2015; Zickfeld et al. 2024). Importantly, it demonstrates that similar processes are present in children as well, extending research showing that obligations and joint commitments influence children's cooperation in “positive,” non‐compromising situations such as helping others in collaborative tasks (Hamann et al. 2012; Tomasello 2020). Similarly, it shows that children have an abstract understanding of the obligations emanating from reciprocal interactions that include cheating behaviors (Wörle and Paulus 2019).

An alternative, although complementary, interpretation is that gratitude motivated children's cheating for their benefactor. Gratitude has been identified as a mechanism driving reciprocity in adults (Tsang 2006) and children from the age of 4 (Beeler‐Duden and Vaish 2020). Interestingly, gratitude has been suggested to elicit general prosocial attitudes and behavior beyond the immediate situation in which the favor was received (i.e., upstream reciprocity; Bartlett and DeSteno 2006; Beeler‐Duden and Vaish 2020), whereas, in the current study, elevated cheating levels in the reciprocity condition were limited to the guessing game and did not extend to the follow‐up die‐rolling game. This null result was unlikely due to children failing to understand how to cheat in the die‐rolling game, as children in both conditions cheated for their own benefit to a similar extent. Instead, once the guessing game was over (and many children reciprocated at least once), children may have considered their debt repaid, requiring no further prosociality in subsequent interactions. While these findings align more closely with the idea that indebtedness motivated children's cheating, additional research is necessary to disentangle these interpretations.

Another possibility is that strategic concerns motivated children's cheating. Instead of feeling obligated to reciprocate a past favor, children might have tried to elicit a future favor from their partner. Indeed, this kind of strategic future‐oriented prosociality has been shown to increase over the age range under study (Grueneisen and Warneken 2022). However, in the current study children were explicitly told when they were playing the last round. Instead of a drop in cheating rates, as would have been expected under a strategic reciprocity hypothesis, we observed the highest level of cheating on the last trial. This pattern is more consistent with the interpretation that children aimed to settle their debts.

### 4.1 Limitations and Future Directions

The current studies have several limitations. In the vignettes of Study 1, children always answered to the experimenter, hence their responses may have been influenced by the desire to be seen as embracing honesty by an adult. However, since children also recognize fulfilling reciprocal obligations as normative (Wörle and Paulus 2019), answering in accordance with honesty norms was not the only obvious normative option. Hence, despite the potential influence of social desirability on children's responses, they still demonstrated an awareness of norms against dishonesty and acknowledged cheating as undesirable even when it helped others. Moreover, children were inclined to cheat themselves to return a favor, further showing that they are willing to override an expectation of honesty.

Furthermore, although we found strong evidence of prosocial cheating to return a favor in the guessing game, this was not the case in the die‐rolling game, which may raise questions about the robustness of the effect. Note, however, that the die‐rolling game differed from the guessing game in important aspects: First, no additional reciprocity manipulation was administered before or during the die‐rolling game. The motivation to return the favor might be confined to the context in which the initial favor occurred rather than resulting in a long‐term, general inclination to break the rules for one's benefactor. Second, because die‐rolls were truly private, we cannot be certain whether individual children cheated. When comparing reported die‐roll outcomes to chance, we did find evidence for cheating in the reciprocity, but not in the control condition, although the condition effect was not confirmed in subsequent analyses. Thus, a fruitful starting point for future research would be to further investigate effects of a reciprocity manipulation in the context of the die‐rolling game (and other cheating paradigms) to examine the generalizability of prosocial cheating to return a favor to other contexts.

The current studies were not designed to directly test the underlying mechanisms of children's tendency to cheat to reciprocate a prosocial act. Future research could systematically test what role obligations and commitments as well as moral emotions such as guilt and gratitude play in children's rule violations to benefit others. Additionally, how children perceive the norm of reciprocity offers an intriguing avenue for future research. Children may consider reciprocity to be a moral obligation that directly competes with other moral norms like honesty. Alternatively, they may view reciprocity as an interpersonal norm that is nevertheless powerful enough to outweigh moral concerns under certain circumstances. To distinguish between these possibilities, future research might ask children to explain the reasons for their dishonest behavior and examine whether they frame reciprocity in explicitly moral terms (e.g., as the right thing to do).

Finally, the current studies were conducted in an urban context in Germany, and this socio‐cultural environment likely influenced both children's evaluations of and their engagement in other‐directed cheating and rule compliance. Cross‐societal studies with adults demonstrate that dishonesty in similar game scenarios is relatively less prevalent in Germany compared to other countries (Cohn et al. 2019; Gächter and Schulz 2016). Similarly, Kanngiesser et al. (2024) found lower (selfish) cheating rates in 7‐ to 12‐year‐old children from Germany compared to children from India. Since culture shapes the extent to which different norms in social dilemma situations are prioritized in adults (Miller and Bersoff 1992) and already in children (Fu et al. 2007), the relative importance of honesty versus reciprocity norms likely differs cross‐culturally, leading to different expected results in the paradigms used in the current studies. A fruitful avenue for follow‐up studies would be to include children from more diverse socio‐cultural backgrounds to gain a more comprehensive understanding of how children navigate norm conflicts.

## 5 Conclusion

Many cooperative interactions are characterized by exchanges of favors over time. But when these favors lead individuals to disregard moral norms, the consequences can be damaging. The present findings reveal that reciprocity, a key mechanism stabilizing cooperation, also carries the potential to compromise honesty from early in development, when children first learn to integrate different norms into their behavior. Children as young as 5 years of age prioritize reciprocal obligations over honesty in certain contexts, suggesting that a tension between moral norms and interpersonal commitments emerges early and constitutes an inherent part of cooperative decision‐making. These results imply that, in addition to supporting cooperation, direct reciprocity also has the capacity to erode moral norms. In this light, children's inclination to return favors may leave them susceptible to exploitation, leading them to break rules they otherwise approve of in the effort to help previous benefactors.

## Author Contributions

**Laura Tietz**: conceptualization, methodology, formal analysis, investigation, data curation, writing—original draft, writing—review and editing, visualization. **Felix Warneken**: conceptualization, methodology, writing—review and editing. **Sebastian Grueneisen**: conceptualization, methodology, formal analysis, resources, writing—review and editing, supervision, project administration, funding acquisition

## Ethics Statement

The studies were reviewed and approved by the Ethics Advisory Board at Leipzig University (approval number: 2022.04.28_eb_150) and meet the requirements of the Declaration of Helsinki. Parental written consent and children's verbal assent were required for participation.

## Conflicts of Interest

The authors declare no conflicts of interest.

## Supporting information




**Supporting Information**: desc70059‐sup‐0001‐SuppMat.docx

## Data Availability

The data and code necessary to reproduce the analyses can be accessed via: https://osf.io/nm8fk/?view_only=90476185c8144d90b12bd3f20da75601.

## Endnotes

1 In our preregistration, we also planned to estimate the mean percentage of children who cheated for the partner in the die‐rolling game using the lying calculator developed by Garbarino et al. (2018). However, at the time of the data analysis, this tool was no longer available online, hence we were unable to run this analysis.
