# Supplementary material for: Children Cheat to Return a Favor
Source: Dev Sci. 2025 Aug 24;28(5):e70059. doi: 10.1111/desc.70059 (PMC12375337; doi:10.1111/desc.70059)
Supplement: Supplementary file 1 — Supporting Information: desc70059‐sup‐0001‐SuppMat.docx [file DESC-28-e70059-s001.docx]

**Supplementary Material**

**List of Abbreviations**

BF Bayes Factor

CI Confidence interval

df Degrees of freedom

GLM Generalized linear model

GLMM Generalized linear mixed model

LM Linear model

LMM Linear mixed model

SE Standard error

1. **Study 1: Vignette evaluations**

**Table S1.1**

*LMMs investigating evaluations of protagonist’s behavior.*

| Predictor | Estimate | SE | 95% CI | χ^2^ | df | *p* |
| --- | --- | --- | --- | --- | --- | --- |
| Full-null comparison |  |  |  | 181.24 | 7 | < .001 |
| Condition (control) * age in months * vignette content (cheating) | 0.044 | 0.022 | 0.000; 0.088 | 4.184 | 1 | .041 |
| Condition (control) * age in months | 0.004 | 0.011 | -0.019; 0.026 | 0.114 | 1 | .735 |
| Age in months * vignette content (cheating) | 0.013 | .0.11 | -0.009; 0.036 | 1.501 | 1 | .221 |
| Condition (control) * vignette content (cheating) | 0.506 | 0.301 | -0.084; 1.096 | 2.995 | 1 | .084 |
| Condition (control) | -0.129 | 0.152 | -0.426; 0.168 | 0.750 | 1 | .386 |
| Age in months | -0.009 | 0.006 | -0.020; 0.002 | 2.582 | 1 | .108 |
| Vignette content (cheating) | -3.250 | 0.152 | -3.547; -2.953 | 172.007 | 1 | < .001 |

*Note.* Reference categories in brackets.

**Table S1.2**

*Follow-up on analysis: LMs investigating evaluations of cheating.*

| Predictor | Estimate | SE | 95% CI | χ^2^ | df | *p* |
| --- | --- | --- | --- | --- | --- | --- |
| Age in months * condition (control) | -0.018 | 0.018 | -0.056; 0.019 | 0.716 | 1 | .299 |
| Age in months | -0.016 | 0.009 | -0.034; 0.003 | 2.099 | 1 | .082 |
| Condition (control) | -0.382 | 0.244 | -0.873; 0.110 | 1.746 | 1 | .111 |

*Note.* Reference category in brackets.

**Table S1.3**

*Follow-up analysis: LMs investigating evaluations of rule compliance.*

| Predictor | Estimate | SE | 95% CI | χ^2^ | df | *p* |
| --- | --- | --- | --- | --- | --- | --- |
| Age in months * condition (control) | 0.026 | 0.013 | 0.000; 0.051 | 1.405 | 1 | .037 |
| Age in months | -0.002 | 0.007 | -0.015; 0.011 | 0.046 | 1 | .713 |
| Condition (control) | 0.124 | 0.173 | -0.225; 0.473 | 0.185 | 1 | .452 |

*Note.* Reference category in brackets. Confidence intervals were computed using the Wald method, resulting in the confidence interval for the significant age*condition interaction including zero.

**Table S1.4**

*Bayesian model comparisons (vignette evaluations – main analysis).*

| Comparison | BF | Evidence level |
| --- | --- | --- |
| Full-null model | 2.333 × 10³¹ | Very strong evidence for the full model over the null model |
| Reduced-full model | 2.599 | Anecdotal evidence for the reduced model over the full model |
| Main effects-reduced model | 176.087 | Very strong evidence for the main effects model over the reduced model |

*Note.* Full model: the three-way interaction between age in months, condition, and vignette content. Reduced model: all two-way interactions between the predictors. Main effects model: main effects of the predictors.

***Alternative follow-up analysis for the significant three-way interaction***

As an alternative way to follow up on the significant three-way interaction between age, condition, and vignette content we analyzed the influence of age and vignette content for each condition separately. In the reciprocity condition, children evaluated cheating significantly more negatively (*M* = 4.29, *SD* = 1.12; *bad*) than rule compliance (*M* = 1.29, *SD* = 0.55; *very good*), χ^2^(1) = 67.14, p < .001. Although the interaction between age and vignette content did not reach significance (χ^2^(1) = 3.65, *p* = .056), this marginally significant effect suggests that with increasing age, children evaluated cheating somewhat less negatively (*b* = -0.02, *SE* = 0.01, *t* = –1.90, *p* = .064), while there was no effect of age on children’s evaluations of rule compliance (*b* = 0.01, *SE* = 0.01, *t* = 0.74, *p* = .465). In the control condition, children evaluated cheating significantly more negatively (*M* = 4.67, *SD* = 0.48; *bad – very bad*) than rule compliance (*M* = 1.17, *SD* = 0.64; *very good*), χ^2^(1) = 118.08, *p* < .001.

**Table S1.5**

*Alternative follow-up analysis: LMMs investigating vignette evaluation in the reciprocity condition.*

| Predictor | Estimate | SE | 95% CI | χ^2^ | df | *p* |
| --- | --- | --- | --- | --- | --- | --- |
| Age in months * vignette content (cheating) | 0.033 | 0.018 | -0.002; 0.068 | 3.654 | 1 | .056 |
| Age in months | -0.007 | 0.009 | -0.025; 0.011 | 0.680 | 1 | .410 |
| Vignette content (cheating) | -3.000 | 0.256 | -3.502; -2.498 | 67.137 | 1 | < .001 |

*Note.* Reference category in brackets.

**Table S1.6**

*Alternative follow-up analysis: LMMs investigating vignette evaluation in the control condition.*

| Predictor | Estimate | SE | 95% CI | χ^2^ | df | *p* |
| --- | --- | --- | --- | --- | --- | --- |
| Age in months * vignette content (cheating) | -0.011 | 0.013 | -0.036; 0.014 | 0.811 | 1 | .368 |
| Age in months | -0.011 | 0.006 | -0.023; 0.001 | 3.16 | 1 | .075 |
| Vignette content (cheating) | -3.500 | 0.160 | -3.813; -3.187 | 118.08 | 1 | < .001 |

*Note.* Reference category in brackets.


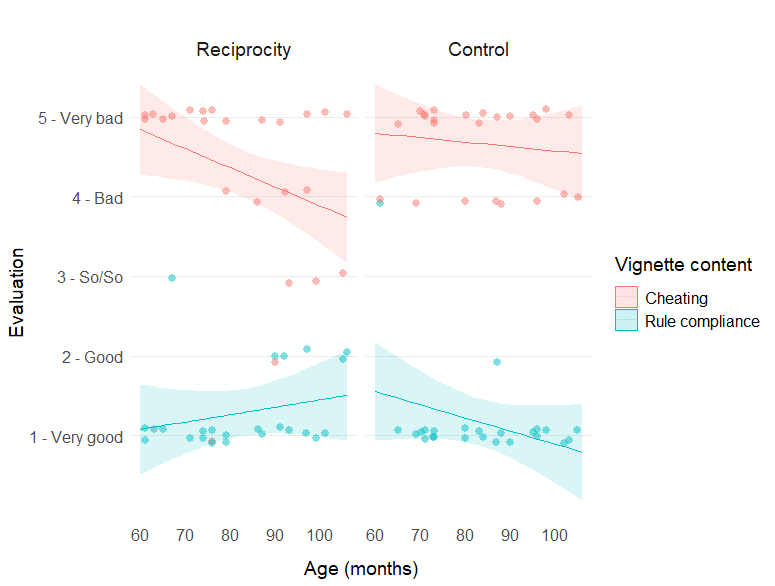
 **Fig. S1.1**. Influence of age and vignette content on children’s evaluations of cheating and rule compliance, separately for each condition (model plot including raw data points). Lines represent model estimates, shaded areas represent 95% confidence intervals.

1. **Study 1: Prosocial explanations**

**Table S2.1**

*Explanations for the protagonist’s cheating provided by participants.*

| Category | *n* | Example |
| --- | --- | --- |
| Prosocial explanations | 17 | “So that Green can have a sticker as well.” |
| Non-prosocial explanations: | 31 |  |
| Wanting to guess correctly | 15 | “So that he knows what it is in the next round” |
| No answer | 6 | “I don’t know” |
| Wanting another sticker | 6 | “I think he wanted two stickers” |
| Wanting to cheat | 2 | “She wanted to cheat” |
| Not wanting the other child to win a sticker | 2 | “So that the other one doesn’t get a dino sticker.” |

*Note.* Reasons participants provided for the vignette protagonist’s cheating. For subsequent analyses, children’s answers were categorized into prosocial explanations (1) and non-prosocial explanations (0).

**Table S2.2**

*GLMs investigating children’s explanation of protagonist behavior.*

| Predictor | Estimate | SE | 95% CI | χ^2^ | df | *p* |
| --- | --- | --- | --- | --- | --- | --- |
| Full-null comparison |  |  |  | 26.008 | 3 | < .001 |
| Condition (control) * age in months | -0.235 | 0.156 | -0.687; -0.007 | 4.159 | 1 | .041 |
| Condition | 1.452 | 0.824 | -0.082; 3.224 | 3.428 | 1 | .064 |
| Age in months | 0.132 | 0.039 | 0.066; 0.220 | 19.547 | 1 | < .001 |

*Note.* Reference category in brackets. When calculating the confidence intervals for the condition*age interaction, several warnings emerged, hence this CI should be interpreted with caution.

**
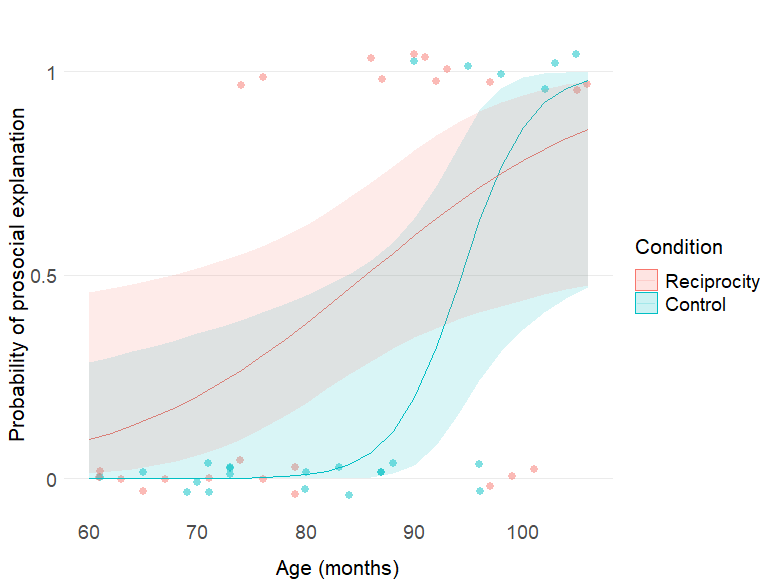
**

**Fig. S2.1**. Influence of age and condition on children’s prosocial explanations for cheating (model plot including raw data points). Lines represent model estimates, shaded areas represent 95% confidence intervals.

**Table S2.2**

*Bayesian model comparisons (prosocial explanations – main analysis).*

| Comparison | BF | Evidence level |
| --- | --- | --- |
| Full-null model | 3.863 × 10^4^ | Very strong evidence for the full model over the null model |
| Full-main effects model | 2.744 | Anecdotal evidence for the full model over the main effects model |

*Note.* Full model: interaction between age and condition. Main effects model: main effects of the predictors.

1. **Study 2: Cheating test 1: Guessing game**

***Main analyses***

**Table S3.1**

*GLMMs investigating cheating in the guessing game.*

| Predictor | Estimate | SE | 95% CI | χ^2^ | df | *p* |
| --- | --- | --- | --- | --- | --- | --- |
| Full-null comparison |  |  |  | 42.419 | 7 | < .001 |
| Condition (control) * trial * age in years | 0.681 | 0.348 | -0.002; 1.363 | 3.968 | 1 | .046 |
| Age in years * condition (control) | 0.421 | 0.645 | -0.844; 1.686 | 1.079 | 1 | .299 |
| Age in years * trial | -0.149 | 0.171 | -0.483; 0.186 | 0.774 | 1 | .379 |
| Condition (control) * trial | 0.541 | 0.378 | -0.199; 1.282 | 2.109 | 1 | .146 |
| Condition (control) | 3.164 | 0.890 | 1.420; 4.909 | 20.115 | 1 | < .001 |
| Trial | 0.713 | 0.192 | 0.337; 1.088 | 15.725 | 1 | < .001 |
| Age in years | 0.006 | 0.312 | 0.606; 0.618 | < 0.001 | 1 | .985 |

*Note.* Reference category in brackets. Confidence intervals were computed using the Wald method, resulting in the confidence interval for the significant condition*trial*age interaction including zero. When computing the two-way interaction models, several warnings emerged, hence these model statistics should be interpreted with caution.

**Table S3.2**

*Follow-up analysis: GLMMs investigating cheating in the guessing game in in trial 1.*

| Predictor | Estimate | SE | 95% CI | χ^2^ | df | *p* |
| --- | --- | --- | --- | --- | --- | --- |
| Condition (control) * age in years | -0.160 | 0.294 | -0.742; 0.415 | 0.298 | 1 | .585 |
| Condition (control) | 0.777 | 0.328 | 0.142; 1.431 | 5.772 | 1 | .016 |
| Age in years | 0.031 | 0.145 | -0.253; 0.317 | 0.047 | 1 | .828 |

*Note.* Reference category in brackets.

**Table S3.3**

*Follow-up analysis: GLMMs investigating cheating in the guessing game in in trial 2.*

| Predictor | Estimate | SE | 95% CI | χ^2^ | df | *p* |
| --- | --- | --- | --- | --- | --- | --- |
| Condition (control) * age in years | 0.242 | 0.294 | -0.334; 0.825 | 0.679 | 1 | .410 |
| Condition (control) | 1.282 | 0.328 | 0.651; 1.940 | 16.309 | 1 | < .001 |
| Age in years | 0.041 | 0.144 | -0.241; 0.325 | 0.083 | 1 | .774 |

*Note.* Reference category in brackets.

**Table S3.4**

*Follow-up analysis: GLMMs investigating cheating in the guessing game in in trial 3.*

| Predictor | Estimate | SE | 95% CI | χ^2^ | df | *p* |
| --- | --- | --- | --- | --- | --- | --- |
| Condition (control) * age in years | 0.391 | 0.280 | -0.153; 0.947 | 1.978 | 1 | .160 |
| Condition (control) | 1.090 | 0.309 | 0.491; 1.706 | 12.906 | 1 | < .001 |
| Age in years | -0.038 | 0.138 | -0.310; 0.233 | 0.076 | 1 | .783 |

*Note.* Reference category in brackets.

**Table S3.5**

*Bayesian model comparisons (cheating in the guessing game – main analysis).*

| Comparison | BF | Evidence level |
| --- | --- | --- |
| Full-null model | 2.183 × 10^8^ | Very strong evidence for the full model over the null model |
| Full-reduced model | 7.770 | Moderate evidence for the full model over the reduced model |
| Full-main effects model | 33.491 | Very strong evidence for the full model over the main effects model |

*Note.* Full model: three-way interaction between age, condition, and trial. Reduced model: all two-way interactions between the predictors. Main effects model: main effects of the predictors.

***Alternative follow-up analysis for the significant three-way interaction***

We alternatively follow up on the significant three-way by testing for the effect of condition and trial for each age group separately (see Fig. S3.1).

In 5-year-olds, we found a significant interaction between condition and trial (χ^2^(1) = 9.83, *p* = .002) indicating that, over the course of the game, 5-year-old’s prosocial cheating increased, while cheating to return a favor remained relatively constant at a higher level (reciprocity condition: *M* = 1.7, *SD* = 1.43, control condition: *M* = 0.96, *SD* = 1.26).

Overall cheating rates were comparably low in 6-year-old children (reciprocity condition: *M* = 1.04, *SD* = 1.33; control condition: *M* = 0.57, *SD* = 0.95), and children’s tendency to cheat increased over the course of the game in both conditions (main effect of trial: χ^2^(1) = 5.37, *p* = .020). However, as models for these two younger age groups revealed inflated estimates, presumably due to the lack of variation in their responses across trials (children tended to either always cheat or never cheat), they should be interpreted with caution.

In 7-year-olds, overall cheating also increased as the game progressed, as indicated by a significant main effect of trial (χ^2^(1) = 4.13, *p* = .042). Children in the reciprocity tended to cheat more than children in the control condition (main effect of condition: χ^2^(1) = 3.38, *p* = .066; reciprocity condition: *M* = 1.26, *SD* = 1.14; control condition: *M* = 0.74, *SD* = 1.05), and this condition difference tended to increase in later trials (condition by trial interaction effect: χ^2^(1) = 3.55, *p* = .060), although both of those effects did not reach significance.

Results further showed that 8-year-old children cheated more in the reciprocity condition than in the control condition, as indicated by a significant main effect of condition (χ^2^(1) = 10.79, *p* = .001; reciprocity condition: *M* = 1.83, *SD* = 1.27; control condition: *M* = 0.74, *SD* = 1.05).

As a robustness check, we additionally compared average cheating across conditions using non-parametric tests. A Wilcoxon rank-sum test confirmed that, on average, children in the reciprocity condition cheated more often (*M* = 1.46, *SD* = 1.31) than children in the control condition (*M* = 0.75, *SD* = 1.08; *W* = 2957.5, *p* < .001). In the reciprocity condition, 37% of children did not cheat at all, while 36.9% of children cheated in all three guessing game trials. In the control condition, 60.9% of children did not cheat at all and only 12% of children cheated in all three trials.

**Table S3.6**

*Alternative follow-up analysis: GLMMs investigating cheating in the guessing game in 5-year-olds.*

| Predictor | Estimate | SE | 95% CI | χ^2^ | df | *p* |
| --- | --- | --- | --- | --- | --- | --- |
| Condition (control) * trial | -9.449 | 2.827 | -14.989; -3.909 | 9.828 | 1 | .002 |
| Condition (control) | 25.585 | 7.292 | 11.293; 39.876 | 17.854 | 1 | < .001 |
| Trial | 3.896 | 2.264 | -0.541; 8.332 | 15.780 | 1 | < .001 |

*Note.* Reference category in brackets.

**Table S3.7**

*Alternative follow-up analysis: GLMMs investigating cheating in the guessing game in 6-year-olds.*

| Predictor | Estimate | SE | 95% CI | χ^2^ | df | *p* |
| --- | --- | --- | --- | --- | --- | --- |
| Condition (control) * trial | 0.6435 | 1.1337 | -1.578; 2.865 | 0.341 | 1 | .560 |
| Condition (control) | 0.975 | 1.875 | -2.701; 4.650 | 0.273 | 1 | .601 |
| Trial | 1.172 | 0.567 | 0.061; 2.283 | 5.373 | 1 | .020 |

*Note.* Reference category in brackets.

**Table S3.8**

*Alternative follow-up analysis: GLMMs investigating cheating in the guessing game in 7-year-olds.*

| Predictor | Estimate | SE | 95% CI | χ^2^ | df | *p* |
| --- | --- | --- | --- | --- | --- | --- |
| Condition (control) * trial | 1.176 | 0.649 | -0.095; 2.448 | 3.546 | 1 | .060 |
| Condition (control) | 1.518 | 0.893 | -0.232; 3.268 | 3.380 | 1 | .066 |
| Trial | 0.607 | 0.312 | -0.004; 1.218 | 4.125 | 1 | .042 |

*Note.* Reference category in brackets.

**Table S3.9**

*Alternative follow-up analysis: GLMMs investigating cheating in the guessing game in 8-year-olds.*

| Predictor | Estimate | SE | 95% CI | χ^2^ | df | *p* |
| --- | --- | --- | --- | --- | --- | --- |
| Condition (control) * trial | 0.855 | 0.696 | -0.508; 2.218 | 1.597 | 1 | .206 |
| Condition (control) | 3.656 | 1.428 | 0.856; 6.455 | 10.795 | 1 | .001 |
| Trial | 0.426 | 0.335 | -0.230; 1.083 | 1.683 | 1 | .194 |

*Note.* Reference category in brackets.


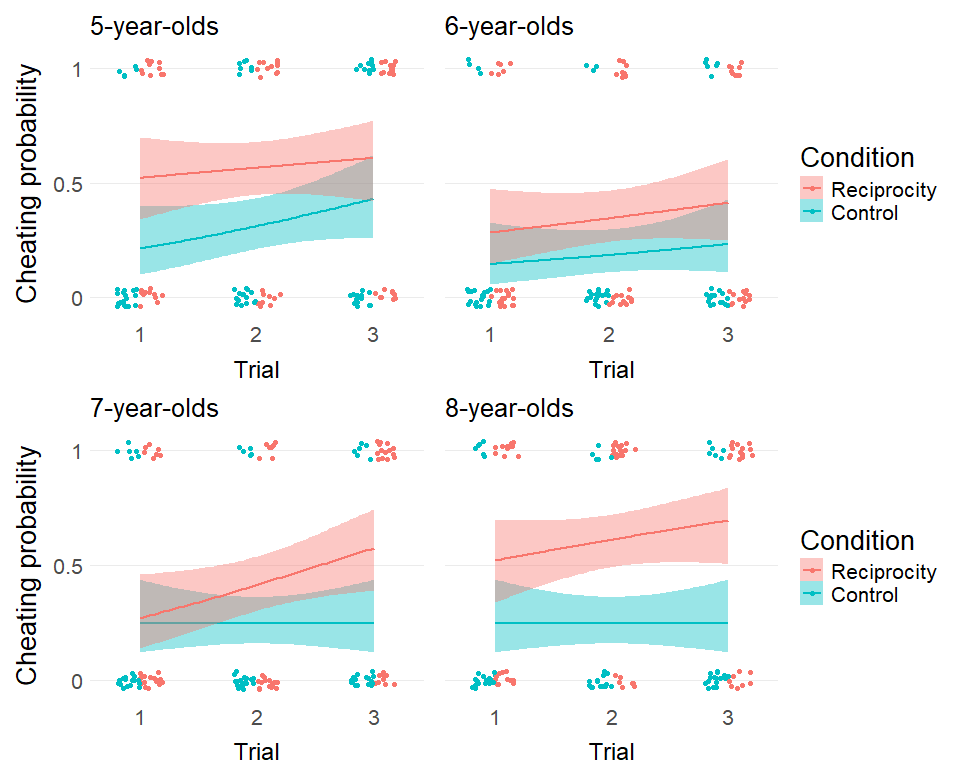


**Fig. S3.1**. Effects of condition and trial on children’s cheating in the guessing game, separately for each age group (raw data plots with raw data points). Shaded areas represent 95% confidence intervals.

1. **Study 2: Cheating test 2: Die-rolling game**

**Table S4.1**

*GLMM investigating dot reports for partner in the die-rolling game.*

| Predictor | χ^2^ | df | *p* |
| --- | --- | --- | --- |
| Full-null comparison | 6.880 | 7 | .442 |

**Table S4.2**

*Bayesian model comparison.*

| Comparison | BF | Evidence level |
| --- | --- | --- |
| Null-full model | 5.680 | Moderate evidence for the null model over the full model |

*Note.* Full model: three-way interaction between age, condition, and trial.

1. **Study 2: Both cheating tests combined**

**Table S5.1**

*GLMMs investigating cheating and reporting a dot, respectively, in both games.*

| Predictor | Estimate | SE | 95% CI | χ^2^ | df | *p* |
| --- | --- | --- | --- | --- | --- | --- |
| Full-null comparison |  |  |  | 32.829 | 7 | < .001 |
| Game partner | -0.259 | 0.204 | -0.660; 0.142 | 4.595 | 1 | .032 |
| Condition (control) * game (die-rolling game) * age in years | 0.146 | 0.565 | -0.962; 1.254 | 0.067 | 1 | .796 |
| Condition (control) * game (die-rolling game) | 2.438 | 0.726 | 1.016; 3.861 | 14.739 | 1 | < .001 |
| Game (die-rolling game) * age in years | -0.047 | 0.281 | -0.598; 0.504 | 0.028 | 1 | .868 |
| Condition (control) * age in years | 0.227 | 0.160 | -0.086; 0.541 | 2.054 | 1 | .152 |
| Condition (control) | 0.460 | 0.183 | 0.100; 0.819 | 6.053 | 1 | .014 |
| Game (die-rolling game) | -0.938 | 0.356 | -1.636; -0.241 | 9.106 | 1 | .002 |
| Age in years | -0.029 | 0.102 | -0.229; 0.171 | 0.081 | 1 | .776 |

*Note.* Reference categories in brackets.

**Table S5.2**

*Follow-up on the interaction: GLMM investigating cheating in the guessing game.*

| Predictor | Estimate | SE | 95% CI | χ^2^ | df | *p* |
| --- | --- | --- | --- | --- | --- | --- |
| Game partner | 0.114 | 0.878 | -1.606; 1.835 | 0.017 | 1 | .896 |
| Condition (control) | 2.804 | 0.763 | 1.309; 4.300 | 19.478 | 1 | < .001 |
| Age in years | 0.038 | 0.369 | -0.685; 0.762 | 0.011 | 1 | .917 |

*Note.* Reference category in brackets.

**Table S5.3**

*Follow-up on the interaction: GLMM investigating dot reports in the die-rolling game.*

| Predictor | Estimate | SE | 95% CI | χ^2^ | df | *p* |
| --- | --- | --- | --- | --- | --- | --- |
| Game partner | -0.347 | 0.245 | -0.828; 0.133 | 2.007 | 1 | .157 |
| Condition (control) | 0.330 | 0.178 | -0.020; 0.679 | 3.430 | 1 | .064 |
| Age in years | -0.030 | 0.102 | -0.229; 0.169 | 0.086 | 1 | .769 |

*Note.* Reference category in brackets.

**Table S5.4**

*Bayesian model comparisons (both cheating tests combined – main analysis).*

| Comparison | BF | Evidence level |
| --- | --- | --- |
| Null-full model | 3.123 | Moderate evidence for the null model over the full model |
| Full-reduced model | 1.677 | Anecdotal evidence for the full model over the reduced model |
| Main effects-reduced model | 34.786 | Very strong evidence for the main effects model over the reduced model |
| Main effects-full model | 219.765 | Very strong evidence for the main effects over the full model |

*Note.* Full model: three-way interaction between age, condition, and game. Reduced model: all two-way interactions between the predictors. Main effects model: main effects of the predictors. When computing the models several warnings emerged, hence these BFs should be interpreted with caution.

1. **Study 2: Exploratory analysis of dot reports for the child**

In addition to the analysis reported in the main text, we also tested whether children’s tendency to report dots for the partner predicted their tendency to report dots for themselves with a GLMM containing an interaction between die-rolling outcome for the partner and condition, main effects of trial and age (in years) as fixed effects, subject number as a random effect as well as the random slopes of trial number nested within subject number.

The full-null model comparison indicated a significant combined effect of the predictors on children’s reports of dots for themselves (χ^2^(5) = 14.09, *p* = .015). Across conditions, the likelihood of reporting a dot for themselves increased with trial number (χ^2^(1) = 8.10, *p* = .004). Children who reported more dots for the partner also tended to report more dots for themselves, but this effect was not significant (χ^2^(1) = 2.97, *p* = .085).

Bayesian model comparisons differed from the frequentist model comparisons, revealing strong evidence for the full model containing the interaction between condition and dot reports for the partner (BF = 21.93). The (non-significant) interaction indicated that in the control condition, children who reported more dots for the partner also reported more dots for themselves (*b* = 0.63, *SE* = 0.29, *z* = 2.15, *p* = .032), while this effect did not exist in the reciprocity condition (*b* = 0.08, *SE* = 0.29, *z* = 0.27, *p* = .788).

**Table S6.2**

*GLMMs investigating dot reports for self in the die-rolling game.*

| Predictor | Estimate | SE | 95% CI | χ^2^ | df | *p* |
| --- | --- | --- | --- | --- | --- | --- |
| Full-null comparison |  |  |  | 14.089 | 5 | .015 |
| Dot report for partner * condition (control) | -0.554 | 0.413 | -1.363; 0.254 | 1.825 | 1 | .177 |
| Dot report for partner | 0.350 | 0.243 | -0.049; 0.749 | 2.972 | 1 | .085 |
| Condition (control) | 0.176 | 0.223 | -0.260; 0.613 | 0.627 | 1 | .428 |
| Trial | 0.326 | 0.116 | 0.099; 0.554 | 8.098 | 1 | .004 |
| Age in years | -0.072 | 0.100 | -0.268; 0.123 | 0.528 | 1 | .468 |

*Note.* Reference category in brackets.

**Table S6.1**

*Bayesian model comparisons.*

| Comparison | BF | Evidence level |
| --- | --- | --- |
| Full-null model | 97.205 | Very strong evidence for the full model over the null model |
| Full-main effects model | 21.933 | Strong evidence for the full model over the main effects model |

*Note.* Full model: interaction between dot report for the partner and condition, as well as main effects of trial and age. Main effects model: main effects of all predictors.

1. **Study 2: Exploratory analysis of effects of reciprocity understanding on children’s cheating to return a favor in the guessing game**

We analyzed if children’s answers in the reciprocity vignettes (i.e., scores ranging from 0 to 4) influenced children’s cheating in the guessing game in the reciprocity condition using LMs. As reciprocity scores were at ceiling in 7- and 8-year-olds (only one 7-year-old and three 8-year-olds did not receive the highest possible score), we only included 5- and 6-year-old children in this analysis. The full-null model comparison revealed only a marginally significant combined effect of the predictors (χ^2^(3) = 11.82, *p* = .092). Hence, we did not conduct any further analyses.

**Table S7.1**

*LM investigating effect of reciprocity understanding on 5- and 6-year-old children’s cheating to return a favor in the guessing game.*

| Predictor | χ^2^ | df | *p* |
| --- | --- | --- | --- |
| Full-null comparison | 11.817 | 3 | .092 |

**Table S7.2**

*Bayesian model comparison.*

| Comparison | BF | Evidence level |
| --- | --- | --- |
| Null-full model | 31.352 | Very strong evidence for the null model over the full model |

*Note.* Full model: two-way interaction between reciprocity score and age in months.
